# Does transition from an unstable labour market position to permanent employment protect mental health? Results from a 14-year follow-up of school-leavers

**DOI:** 10.1186/1471-2458-8-159

**Published:** 2008-05-13

**Authors:** Ieva Reine, Mehmed Novo, Anne Hammarström

**Affiliations:** 1 Family Medicine, Department of Public Health and Clinical Medicine, Umeå University, SE-901 85 Umeå, Sweden

## Abstract

**Background:**

Having secure employment, in contrast to being unemployed, is regarded as an important determinant of health. Research and theories about the negative health consequences of unemployment indicated that transition from unemployment to a paid job could lead to improved health. The objective of this study was to test the hypothesis that obtaining permanent employment after being in an unstable labour market position protects mental health.

**Methods:**

A 14-year follow-up of all graduates from compulsory school in an industrial town in northern Sweden was performed at ages 16, 18, 21 and 30 years. Complete data on the cohort were collected for 1044 individuals with the aid of a comprehensive questionnaire. The response rate was 96.4%. The health measurement used in this study was the psychological symptoms analysed by multivariate logistic regression. Those who obtained permanent employment were the focus of the analysis. This group consisted of people who were in an unstable labour market position for a year or more between the ages of 25 and 29, and who had acquired a permanent job one year before and at the time of the investigation.

**Results:**

After controlling for gender as well as for an indicator of health-related selection, possible confounders and mediators, an association was found between the lower probability of psychological symptoms and obtaining permanent employment (OR = 0.35, 95% CI 0.19–0.63) as well as having permanent employment (OR = 0.22, 95% CI 0.10–0.51).

**Conclusion:**

Our findings suggest that transition from an unstable labour market position to permanent employment could be health-promoting, even after controlling for possible confounders and mediators, as well as for an indicator of health-related selection. However, as there are few studies in the field, there is a need for more longitudinal studies in order to further analyse the relationship and to examine possible explanations. The policy implication of our study is that the transformation of unstable labour market positions into permanent employment could contribute to better public health.

## Background

The importance for the health situation of having secure employment has been increasingly emphasised in new public health policies, and having a job, in contrast to being unemployed, is regarded as an important determinant of health [1,2]. A possible explanation, based mainly on research and theories about the negative health consequences of unemployment, is that transition from unemployment to a paid job improves health.

The first meta-analysis about the possible health effects of reemployment was published recently and showed improvement in mental health when unemployed persons were reemployed [3]. Other research has suggested that the positive effects of reemployment after unemployment may be limited to those who gain satisfactory new jobs [3-5]. A recent German study on health satisfaction found that even though there is a negative health effect of long-term unemployment on both men and women, reemployment has a positive effect on health satisfaction independently of the duration of the period of unemployment for both men and women [6]. Overall, research in the field is scarce.

There is more abundant research on, and agreement about, the relation between unemployment on the one hand and poor well-being and poor psychological and physical health on the other [3,6-11]. The longer the time in unemployment [3,6] and the younger the ages [3,12], the worse are the consequences to health. Different theoretical models have been used to explain the association between unemployment and health and these models can be indirectly used to explain the possible health-promoting effects of reemployment after being unemployed. *The economic deprivation model *assumes that unemployment leads to poverty and other forms of disadvantage, which we know are associated with poor health. Research has also shown a negative relationship between perceived financial strain and well-being during unemployment [13,14]. According to the *theory of latent function*, there are also non-financial benefits provided by a job, such as opportunities for social contacts and improved status (for example, that people do not look down on you) [15].

On the other hand, the health situation of the unemployed may not be improved by reemployment. A previously long-term unemployed person who is reemployed may continue to have the self-image and identity of an unemployed person (e.g. feeling that people look down on you) [16]. Moreover, insecure employment with a potential risk of unemployment could be health-impairing [17,18]. It was found that those who are permanently employed in non-preferred occupations and undesired workplaces reported more symptoms of ill-health than a comparative group [19]. Also, those who were not in preferred occupations continued to cope with job loss similarly to those who were unemployed [20].

Different models have been suggested for how a stressor may affect health over time. The *model of accumulation of strain *implies that strain does not go away when the stressor disappears [21]. According to the Karasek-Theorell's *demand-control model*, unemployment can be regarded as a passive situation, with negative health consequences related to a combination of low control and low demands [22]. The strain of being unemployed may persist when reemployed.

Moreover, obtaining a job can be a new stressor that adds additional strain to the strain accumulated during unemployment. The combination of employment and family demands (such as having dependent children) could make reemployment after unemployment more stressful, especially for mothers who usually have the main responsibility for domestic work [23,24]. The value of having a job, commonly measured by the Work Involvement Scale [25], may also have an impact on well-being in the transition from unemployment to work [9,26]. Long-term unemployment may lead to decreased value in having a job [27-29] which can influence the reemployment process negatively.

Among demographic variables, gender was found to have interesting patterns, e.g. that it might be more difficult for women with good health to enter paid employment than men with a similar health situation [30]. Simultaneously as a meta-analysis concludes that unemployment is psychologically more damaging for women [3], it has also been found that transition from unemployment to a paid job was only associated with improved psychological well-being among women [31]. Therefore, more research is needed about the importance of gender in the transition from unemployment and other kinds of unstable labour market positions to employment.

To summarize, the question of whether transition from an unstable labour market position to permanent employment protects mental health remains to be studied.

There are several methodological inadequacies in previous research on the health consequences of reemployment. First, possible confounders in the relationship between reemployment and health need to be taken more into account. Having employed rather than unemployed relatives, especially in younger ages, might increase the possibilities of finding a job. Socio-economic status may also act as a confounder between health status and reemployment. There is overwhelming documentation of the relation between a working-class position and poor health status [32]. Also, socioeconomic status can influence reemployment because reemployment may have more financial importance among blue-collar workers, while reemployment may mean the status of having a job among white-collar workers [33,34].

Second, most of the studies have a design that makes it impossible to control for health-related selection due to lack of information about the health status before entering the labour market. Health selection means that early health status could have an impact on adult health status [35]. Poor health before entering the labour market could lead both to difficulties in obtaining a job and to continued poor health if reemployed [3,36]. Earlier research has also shown that a history of poor health is associated with a higher risk of mobility out of employment and a lower chance of mobility into employment for both men and women [37].

Third, the labour market during the last two decades has become increasingly polarised between two extremes – "the secure centre", characterised by permanent employees, and the "insecure periphery", characterised by different kinds of temporary employment [38] as well as by unemployment and labour market programmes [17]. Thus, the frontier between employment and unemployment is becoming blurred and it is methodologically difficult to obtain accurate data about exposure [39]. It has therefore been suggested that studies on the labour market position should move away from the comparison between employed and unemployed towards a more dynamic comparison of workers with stable versus unstable jobs [10,39]. As young people often move between different temporary situations such as unemployment, labour market programmes, studies and precarious employments [7] the analysis of an unstable labour market position rather than unemployment alone would give a more comprehensive picture of the transition to permanent employment.

Fourth, the non-response rate is often high in longitudinal studies which are necessary for analysing health changes related to labour market trajectories. A high response rate is crucial for including marginalised people who are often missing in other studies, and earlier research has shown that even a low non-response rate can introduce serious bias and uncertain results [40].

Thus, there is a strong need for longitudinal studies with high response rates, starting at a young age before the cohort enters the labour market, so that health-related selection can be controlled for.

The aim of this study was to analyse whether transition from an unstable labour market position to permanent employment could protect mental health. The hypothesis was that obtaining permanent employment after being in an unstable labour market position is associated with a lower risk of psychological symptoms after controlling for gender, possible confounders and mediators as well as an indicator of health-related selection.

## Methods

The study was carried out in a medium-sized industrial town in the north of Sweden. The cohort, consisting of all 1083 pupils (506 girls and 577 boys) aged 16 who attended or should have attended the last year of compulsory school in 1981, was followed up at the ages of 16, 18, 21 and 30. Extensive work was carried out to reach every participant, including those who had moved, in order to minimize the non-response rate to a minimum [8]. Complete data for the whole 14-year follow-up period were collected for 1044 individuals (547 men and 497 women), i.e. 96.4% of the original sample.

Data were mainly collected with comprehensive questionnaires during school hours (at the ages of 16 and 18) and during class reunions (at the ages of 21 and 30). The questionnaires covered the following main themes: psychological and physical symptoms, health behaviour, experiences of work and unemployment, as well as socio-economic data. The questionnaires, including a reminder, were sent to those who could not attend these reunions. Participants who failed to reply were contacted by telephone and, if they agreed to participate, a telephone interview was performed.

A within-subject design was used, based on three repeated measurements. The surveys from 1981 (when the respondents were 16 years old), 1986 (21 years) and 1995 (30 years) were used in this article. The method is extensively described elsewhere [35,40].

### Statistics

Windows version 15.0 of SPSS was used for data analysis. We assessed the distribution of the dependent variable (health outcome) and independent variables between the three groups of labour market position for men and women separately (Tables 1a and 1b). A chi-square test was used to analyse differences for the dichotomous variables between the studied groups in Tables 1a and 1b. A p-value <0.05 was chosen as statistically significant. The correlations between all independent variables included in the analyses were calculated [see Additional file 1]. The internal consistency for all indices included in the model was measured by Cronbach's alpha [see Additional file 2].

**Table 1:** 1a. Distribution of the confounders and mediating variables included in the multiple regression analyses among men without permanent employment, obtained permanent employment and in permanent employment (percentages and p-values).

				p	p	p
	Without permanent employment	Obtained permanent employment	Permanent employment	Without permanent employment/obtained permanent employment	Without permanent employment/permanent employment	Obtained permanent employment/permanent employment
	%	%	%			
	n = 42	n = 46	n = 324			
*Dependent variable*						
Psychological symptoms (age 30)	52.4	13.0	20.1	0.687	0.409	0.965
*Independent variables*						
Psychological symptoms (age 16)	38.1	21.7	20.1	0.513	0.453	0.873
Unemployed at young age (16–21)	37.7	10.9	10.2	<0.001	<0.001	0.894
Have children (21)	4.8	2.2	6.8	0.612	0.605	0.221
Financial problems (21)	66.7	47.8	42.0	0.075	0.002	0.452
Unemployed relatives (21)	16.7	2.2	7.7	0.012	0.044	0.194
High WIS (30)	21.4	28.3	28.7	0.477	0.324	0.950
Blue-collar worker (30)	64.3	45.7	43.5	0.079	0.011	0.785
Do not have a chance to do what I would prefer to do (30)	66.7	52.2	43.8	0.172	0.005	0.286
High demands (30)	21.4	45.7	31.8	0.015	0.176	0.060
Low control (30)	45.2	32.6	26.2	0.190	0.011	0.370
Poor social network (30)	11.9	30.4	32.4	0.006	0.058	0.784
Poor social support (30)	42.9	26.1	22.8	0.005	0.070	0.633
Risk of unemployment (30)	81.0	47.8	25.9	0.001	<0.001	0.002
People look down on me (30)	40.5	32.6	20.7	0.385	0.005	0.075

**1b. Distribution of the confounders and mediating variables included in the multiple regression analyses among women without permanent employment, obtained permanent employment and in permanent employment (percentages and p-values).**						

				p	P	p
	Without permanent employment	Obtained permanent employment	Permanent employment	Without permanent employment/obtained permanent employment	Without permanent employment/permanent employment	Obtained permanent employment/permanent employment
	%	%	%			
	n = 31	n = 26	n = 248			

*Dependent variable*						
Psychological symptoms (age 30)	54.8	26.9	26.6	0.002	0.007	0.134
*Independent variables*						
Psychological symptoms (age 16)	41.9	53.8	37.1	0.176	0.948	0.138
Unemployed at young age (16–21)	41.9	7.7	11.7	<0.001	<0.001	0.569
Have children (21)	6.5	3.8	11.7	0.750	0.371	0.216
Financial problems (21)	74.2	50.0	58.1	0.065	0.086	0.426
Unemployed relatives (21)	22.6	7.7	7.7	0.052	0.007	0.996
High WIS (30)	9.7	19.2	32.7	0.426	0.008	0.149
Blue-collar worker (30)	38.7	30.8	36.7	0.537	0.827	0.552
Do not have a chance to do what I would prefer to do (30)	58.1	34.6	44.0	0.077	0.137	0.363
High demands (30)	29.0	30.8	38.7	0.893	0.285	0.427
Low control (30)	41.9	38.5	25.4	0.771	0.054	0.159
Poor social network (30)	16.1	36.6	31.0	0.130	0.088	0.705
Poor social support (30)	29.0	30.8	30.6	0.888	0.855	0.990
Risk of unemployment (30)	83.9	50.0	29.8	0.005	<0.001	0.003
People look down on me (30)	48.4	30.8	28.1	0.151	0.025	0.822

Sensitivity analyses were performed for both the dependent and the independent variables with ordinal scales in order to define how to dichotomise variables. Thus, different cut-off points of each specific variable were tested in the regression analyses. In all cases the results were the same in the sensitivity analyses and then the guiding principle was to dichotomise at the 75^th ^percentile.

In order to analyse whether the transition from an unstable labour market position to permanent employment was associated with the lower probability of having psychological symptoms, multivariate logistic regression analysis was used to estimate the odds ratio (OR) with 95% confidence intervals (CI) for the health outcome in relation to the variable "labour market position" after control for gender, possible confounders and mediators as well as for the indicator of health-related selection (Table 2). Bivariate regression analyses (not shown in the Table) were performed for the variable "labour market position" and the outcome variable in order to analyse the change in OR between the bivariate and the multivariate regression analyses. Possible interaction between gender and labour market position was assessed with multiplicative interaction.

**Table 2:** Multivariate logistic regression for psychological symptoms (75th percentile) in relation to labour market position after controlling for the indicator of health-related selection as well as possible confounders and mediators [odds ratios (OR), 95% confidence intervals (95%CI)].

	OR	95% CI
*Labour market position between age 25 and age 30*		
Without employment (Reference)	1	
Obtained employment	0.35	0.19–0.63
Permanent employment	0.22	0.10–0.51
Gender	0.93	0.63–1.36
Psychological symptoms (16)	2.18	1.48–3.23
Unemployed at young age (16–21)	1.26	0.74–2.15
Have children (21)	0.92	0.46–1.83
Financial problems (21)	1.47	0.99–2.17
Unemployed relatives (21)	1.19	0.62–2.25
Blue-collar worker (30)	0.67	0.44–1.02
Do not have a chance to do what I would prefer to do (30)	1.76	1.19–2.61
High WIS (30)	1.01	0.66–1.55
High demands (30)	1.57	1.07–2.31
Low control (30)	0.79	0.50–1.24
Poor social network (30)	0.71	0.46–1.10
Poor social support (30)	1.45	0.97–2.18
Risk of unemployment (30)	1.04	0.69–1.59
People look down on me (30)	2.01	1.33–3.04

The model Chi-square was significant at p = <0.001 (x^2 ^= 112.02, DF = 17). The explained variance in the regression models calculated by Cox & Snell R Square was 0.14).

Written consent to participate was given by the participants in the study. The Ethics Committees of Umeå and Uppsala Universities approved the study.

### Outcome variable

The outcome variable *psychological symptoms *at age 30 was measured with a well-known and validated scale from the Swedish Survey of living conditions [41,42]. The scale consisted of questions about symptoms during the last year with the answer alternatives "yes" (coded as 1) or "no" (coded as 0). The index of psychological symptoms included six items on restlessness, concentration problems, nervousness, palpitations, anxiety and other nervous symptoms. The range of the index was from 0 to 6, with higher values corresponding to more psychological problems.

The proportion over the cut-off point (≥ 1) was defined as those with psychological symptoms. Those below the cut-off point (<1) were defined as not having psychological symptoms.

### Independent variables

#### Labour market position

The variable "labour market position" was measured with a specially constructed battery of questions where the participants were asked to report how long they had been unemployed, employed, studying, and participating in labour market programmes, etc., since the last follow-up. The reports were done for each school-year term (autumn and spring) during the period between 1986 and 1995. If the information about the labour market programmes and unemployment was missing or incomplete, it was supplemented with data from the Swedish Labour Market Board's register.

An unstable labour market position was defined as being in unemployment, occasional jobs or labour market programmes. The aim of the labour market programmes in Sweden during the 1990s was to provide the unemployed with work experience and training in the regular labour market. A "vocational training" aimed at enhancing mobility. Matching on the labour market was also a part of the labour market programmes, and provided qualified theoretical or practical education, which was either purchased from educational enterprises or the regular education system [43,44].

Since we were interested in the transition from an unstable labour market position to employment, the population was classified into three types of labour market groups. Through fulfilling the three criteria – a), b) and c), groups with the highest exposure to different kinds of labour market positions were defined.

1. Reference group (without permanent employment): a) ≥ one year of an unstable labour market position between age 25 AND 29 and b) unstable labour market position for the last year prior to the investigation AND c) being in an unstable labour market position at the time of the investigation at age 30 (n = 73).

2. Obtained permanent employment: a) ≥ one year of an unstable labour market position between age 25 and 29 AND b) having permanent employment one year prior to and c) at the time of the investigation at age 30 (n = 72).

3. Constant permanent employment: a) < one year of an unstable labour market position between age 25 and 29 AND b) having permanent employment one year prior to and, AND c) having permanent employment at the time of the investigation at age 30 (n = 572).

Those who did not belong to any of these three groups were excluded from the analyses (n = 322).

The variable "labour market position" was defined independent of having part-time or full-time employment.

#### Other independent variables

Gender was defined as 1 = men, 0 = women.  The other independent variables were defined as potential *confounders *(i.e. variables associated with both the dependent and the independent variables) or potential *mediators *(i.e. variables that interplayed between the dependent and the independent variables). Those variables that could not be defined as either confounders or mediators were defined as having an *unclear role*.

##### Indicator of health-related selection at age 16

As indicator of health-related selection we used *psychological symptoms at age 16*, which was measured with the same questions as those in the index of psychological symptoms at age 30 (and dichotomised in the same way).

##### Potential confounders at age 21

Unemployment was measured with the same battery of questions as the variable "labour market position". Those who were without a job for at least 6 months between ages 16 and 21 were defined as unemployed at a young age (= 1).

The variable *have children *was measured with one question and defined as those having at least one child (= 1).

*Financial problems *(cash margin) was measured with one question about the possibility of obtaining a certain amount of money (corresponding to 500 euros) within a week by one's own means, with alternative responses yes (= 0) or no (= 1).

*Unemployed relatives *was measured with one question (yes = 1, no = 0) about having an unemployed person in the family during the last 12 months.

##### Potential confounders, mediators and factors with an unclear role at age 30

Socio-economic position was measured with one question about the subject's own occupation and defined according to the Swedish socio-economic (SEI) classification of occupational categories [45]. *Blue-collar worker *(or manual worker) was coded as 1 and white-collar worker (or non-manual worker) was coded as 0 (confounder).

A negative answer to the question "Do you do what you want to do?" regarding type of occupation (work, studies, unemployment etc.) was considered to reflect *not having the chance to do what one would prefer to do *(= 1) (possible mediator) [46].

The psychosocial need for a job was measured by the Work Involvement Scale (WIS) which was developed and validated by Warr, Cook and Wall [25]. The WIS-scale consisted of six seven-grade questions about work commitment with a range from 6 to 42. High scores indicated high work involvement. The proportion over the cut-off point was defined as *high work involvement *(unclear role).

Karasek-Theorell's two dimensional model was used to construct the indices of demands and control [22] in relation to the current occupational situation (including employment, studies, unemployment, etc.). The index of *high demands *was measured from six questions with four answer alternatives each (giving a range of the index from 6 to 24) on qualitative and quantitative demands (referring to quantity of work, intellectual requirements, and time constraints of the job). The index of *low control *(referring to the possibilities of making decisions, being creative and using and developing one's own skills) was measured in the same way as high demands. The proportion over the cut-off point was defined as those with *high demands *and *low control *correspondingly (possible mediators).

Validated questions on social network and social support indices developed by Henderson, Duncan-Jones, Byrne and Scott were used [47]. *Poor social network *was measured with four questions (each with six answer alternatives) about quantitative social network (range 4 to 24). *Poor social support *(material and emotional) was measured with six questions (with four answer alternatives) about qualitative aspects of social support (range 6 to 24). The proportion above the cut-off point was defined as those with poor social support and poor social network correspondingly (unclear role).

*Risk of unemployment *was measured with the question "How big is the risk that you can involuntarily become unemployed?" The question had four answer alternatives (1 = high, 4 = no risk) and was dichotomised as *yes *if the answer was high or some risk (= 1) and no if low or no risk (= 0) (possible mediator).

The question "*People look down on me*" had a 7-grade scale from disagree to completely agree [35]. The scale was dichotomised at the 75^th ^percentile. The proportion above the cut-off point was defined as those who felt that others look down on them (= 1) (possible mediator).

Many other possible confounders, such as education and indicators of externalising problems (such as alcohol consumption and use of narcotics) could have been included in the analyses. However, due to the risk of multicollinearity the number of possible confounders had to be kept low. For example, the education variable was excluded from the model due to its high correlation with social class among women (0.480).

## Results

Table 1a and 1b show the distribution of the dependent and independent variables among men and women separately in different labour market positions, i.e. among those who were without permanent employment, had obtained permanent employment and had permanent employment.

Among women, but not men, significant differences in the psychological symptoms at age 30 were found between those without permanent employment compared to those who had obtained a permanent job as well as those with permanent employment. Overall, those men (and to a lesser extent women) without permanent employment were worse off than those with permanent employment and those who had obtained a permanent job. With one exception (*risk of unemployment*) the situation of those who had obtained a permanent job did not differ significantly from those who had permanent employment.

Gender differences were tested within the three groups of labour market position between Tables 1a and 1b, and only found for social class among those without permanent employment, where more men than women were of low social class (p = 0.031). Among those in permanent employment, women had more often children (p = 0.024) and financial problems (p = <0.001) as well as poorer psychological health at both age 16 and 30 (p = 0.005) compared to men. Women who became permanently employed had poorer psychological health at both ages 16 and 30 than men (p = <0.001).

A separate analysis of labour market activities among those without permanent employment at age 25, i.e., during the first year of the exposure period for the variable "unstable labour market position", showed that 32 percent were unemployed, 15 percent had occasional jobs, 14 percent were in labour market programmes while the rest were in studies or in permanent employment (data not shown in the tables). As regards the excluded group, the majority had permanent jobs (62 percent), while 25 percent were studying.

The correlations between labour market position, psychological symptoms at age 16 as well as all confounders and mediators included in the model (p = <0.05) are shown in Additional file 1. The highest correlations were found between labour market position and risk of unemployment among both men (0.362) and women (0.344).

A multivariate logistic regression was performed in order to analyse whether the transition from an unstable labour market position to permanent employment was associated with psychological symptoms. The results are presented in Table 2.

The multivariate analysis showed that the odds ratio for the psychological symptoms was significantly lower in the groups obtaining permanent employment and with permanent employment, compared to the group without permanent employment. Also, psychological symptoms at age 16, not having the chance to do what one would prefer to do, high demands and "people look down on me" had strong associations with psychological symptoms at age 30.

A separate bivariate analysis was performed to analyse the association between psychological symptoms at age 30 and the variable labour market positions (data not shown in the tables). For those who obtained permanent employment the odds ratio for psychological symptoms was 0.26 (95% CI 0.16–0.43) and for those in permanent employment the odds ratio was 0.19 (95% CI 0.09–0.41). Compared with the results in Table 2, there were no major changes of the OR for permanent employment, while the OR for obtaining permanent employment diminished.

Two other indicators of health-related selection were also tested – psychological health (measured with questions identical to those used at age 16 and at age 30 and dichotomised in the same way) at age 18 and at age 21. If health-related selection was measured at 18 respectively at age 21 the OR for having lower scores of psychological symptoms at age 30 among those who obtained permanent employment was 0.34 (95% CI 0.18–0.63) respectively 0.39 (95% CI 0.21–0.74). Also, if the health-related selection was measured at 18 respectively at age 21 the OR for having lower scores of psychological symptoms at age 30 and having permanent employment was 0.19 (95% CI 0.08–0.47) respectively 0.22 (95% CI 0.09–0.55). Similarly to age 16, there was a strong association between the presence of psychological symptoms at age 30 and the indicators of health-related selection at age 18 (OR = 1.35, 95% CI 1.21–1.51) and age 21 (OR = 1.36, 95% CI 1.24–1.50).

Multiplicative interaction analysis was run in order to test for interactions between gender and labour market position. No significant interaction effects were found, even when controlled for all other independent variables in the model.

Other possible confounders (education, alcohol consumption, smoking, use of narcotics) were also tested to be included in the multivariate regression analyses. None of them changed the significant association between psychological symptoms and labour market status.

The model Chi-square was significant at p = <0.001 (x^2 ^= 112.02, df = 17). The explained variance in the regression models, calculated by Cox & Snell R Square, was 0.14.

## Discussion

The results showed that even after controlling for all the independent variables, including the indicator of health-related selection, a positive association was found between obtaining permanent employment after an unstable labour market position and a lower probability of having psychological symptoms.

### On the method

The attrition rate in this study was as low as 3.6 per cent, which may be due to both the Swedish system of personal identification numbers, which makes it possible to find the address of almost anyone who lives in the country, and the fact that much effort had been expended to find even those who are usually left out in other studies [40].

Structural changes in the labour market in Sweden, including increasing unemployment and increasing work demands in all sectors, took place in the mid 1990s. Thus, we could assume that those who gained permanent employment during these conditions might be positively health-selected into employment. Those who remained unemployed could be selected out of the labour market due to poor health. However, the longitudinal design of the study made it possible to control for the health-related selection, i.e. the health situation at age 16 before entry into the labour market.

Several possible limitations of our study need to be discussed. A problem when analysing indicators of health-related selection is which time is best for measuring it. At age 16 the cohort participants were still in compulsory school and thus had not yet been exposed to the labour market. But the late teenage years and early adulthood is the period of greatest risk for the onset of affective disorders and therefore it might have been more appropriate to use health at the age of 18 or 21. However, at those ages the cohort had already been exposed to the labour market which could have affected their health status. For example, half of the cohort had experienced unemployment at age 21 and earlier research has shown the negative health consequences of unemployment already at an early age such as 18 [48] and 21 [7,8,27]. We tested health-related selection also at the ages of 18 and 21, but the lower probability of psychological symptoms among those who had obtained permanent employment and among those having permanent employment remained at both ages 18 and 21. Thus, we chose to consider health at age 16 as the main measure of health-related selection.

As young people are generally healthy, the probability is low that medical diagnoses will appear as a consequence of an unstable labour market position. Therefore, it seemed accurate to use self-reported symptoms. Moreover, it was shown that self-reported symptoms are predicative of future medical diagnosis [49]. Due to the young age of the cohort, the number of persons with high scores in the measure of psychological symptoms is small, and the cut-off point for psychological symptoms was set at the 75^th ^percentile in order to detect a group at risk.

Another possible limitation of our study is the dichotomisation of the variable psychological symptoms at age 16. The reason for dichotomisation of the variable was that it should be measured in the same way as the identically measured outcome variable (i.e., psychological symptoms at age 30) which had to be dichotomised in the logistic regression analyses. Moreover, separate analyses showed that the association between lower scores of psychological symptoms at age 30 and obtaining permanent employment as well as having permanent employment remained significant even if the indicator of health-related selection variable was used as a continuous variable.

A further limitation is that the labour market position variable was measured from age 25, but we have no measure of the health situation or life circumstances at this age. The health of the cohort was measured with quite long intervals (age 16, age 18, age 21 and age 30). Therefore, the discussion of possible confounders in our study has to be hypothetical. The problem of attributing psychological health at age 30 to changes in the labour market position during a relatively long time period has been addressed by limiting the time period of the labour market position to five years (from age 25 until age 29) rather than to ten years.

In Sweden the trial period for obtaining permanent employment is six months. Therefore, among those who obtained employment, one year of permanent paid work was considered to be a valid measure of firm establishment in the labour market.

A question that needs to be raised is whether the group "without permanent employment" mainly consisted of unemployed. If so, our study would not contribute to the already existing evidence on reemployment after unemployment and health. However, the group did not consist mainly of the unemployed, but had an unstable labour market position in a broader perspective.

The multivariate analysis presented in this study was controlled for some important confounders. Nevertheless, there are other possible confounders which were tested (such as level of education, substance use and other indicators of externalising problems) that may influence one's psychological health during this time period. None of them changed the significant association between psychological symptoms and labour market status. In addition, the number of independent variables must be kept low in order to decrease the risk of mass significance. The importance of unemployment and social exclusion for health has also been more and more emphasised, for example in the WHO Commission of Social Determinants of Health [50]. Labour market position has been shown to be a key determinant of health [2,50,51]. Our findings regarding the importance of labour market programmes for health are in accordance with those statements, even though it is never possible to take all possible confounders into account.

The multivariate analysis model was tested separately for men and women, which gave similar results, indicating that obtaining employment was important to both men and women. Therefore, in order to increase the power and avoid the risk of Type II errors due to a small number of subjects in the subgroups, we chose to adjust for gender in the multivariate analysis.

### On the results

In relation to psychological symptoms we confirmed our hypothesis that reemployment after being in an unstable labour market position was associated with a lower probability of having psychological symptoms. Thus, our study indicated that the transition from an unstable labour market position into permanent paid work seemed to improve psychological health among both men and women. This trend is also found in some of the other available studies in the field [3,5,6]. A longitudinal study of the German population shows that reemployment has a positive effect on health satisfaction for both men and women [6].

Of the analysed possible confounders, we did not find any significant association with psychological symptoms in the multivariate regression. Our results are supported by a German study of reemployment [6] where none of the included confounders, except having children among women, was found to have an effect on the dependent variable (health satisfaction).

Overall, the association between obtaining permanent employment and psychological symptoms was not influenced substantially by including the possible mediators in the multivariate analyses. However, three possible mediators seemed to be related to the dependent variable.

The strong association between health outcome and inability to do what a person wanted could be explained by the fact that people in unstable labour market positions may be expected to take any available job. Moreover, studies on labour market conditions indicate the negative health effects of being "locked-in" at non-preferred and undesired work [19].

The multivariate analysis showed strong association between high demands and the psychological symptoms. High demands in a newly obtained job can be a new stressor that adds additional strain to the strain accumulated during unemployment. From a theoretical point of view our results may be interpreted as if the high demands related to getting permanent employment among men were worse than the exposure to strain in an unstable labour market position. This finding is in concordance with another Swedish study indicating that the psychosocial work environment could be worse in permanent jobs than in temporary jobs, especially with regard to higher demands and more stress [17]. However, our study could not verify that the other variable in the strain model – low control, was related to the outcome variable [21].

In accordance with the theory of latent functions [15], obtaining permanent employment could lead not only to a better economic situation but also to improved self-esteem. However, the association between the psychological symptoms and a feeling of being looked down upon suggests that this may not be the case. On the other hand, those who obtained permanent employment did not report significantly higher experience of being looked down upon than those in permanent employment (Tables 1a and 1b). It seemed that the studied measure of self-esteem might operate in other contextual situations rather than in obtaining a job.

Earlier research gives strong evidence for associations between risk of unemployment and psychological symptoms [38]. In our multivariate analyses there was no association between risk of unemployment and psychological symptoms. However, most earlier studies have not controlled for confounders [38]. The lack of associations in our study could also depend on the correlations between risk of unemployment and the variable labour market positions.

## Conclusion

The transition from an unstable labour market position to permanent employment seemed to be health-promoting, even after controlling for possible confounders and mediators as well as an indicator of health-related selection. Our results, therefore, suggest that there might be more complex underlying mechanisms that influence the well-being of those who had obtained employment. However, as there are few studies in the field there is a need for more longitudinal studies in order to further analyse the relationship as well as possible explanations. The policy implication of our study is that transformation of unstable labour market positions into permanent employment could contribute to better public health.

## Competing interests

The authors declare that they have no competing interests.

## Authors' contributions

IR conceived the paper topic, performed the data analyses and drafted the manuscript. MN participated in the revision of the article. AH designed and coordinated the study and revised the manuscript. All authors have read and approved the final version of the manuscript.

## Pre-publication history

The pre-publication history for this paper can be accessed here:



## Supplementary Material

Additional file 1 Correlations between the variables labour market position, health-related selection, possible confounders and mediators among men (below the empty cells and italics) and women (above the empty cells). The data provided represent correlations between the independent variables. Click here for file

Additional file 2 The internal consistency for all indices included in the model measured by Cronbach's alpha (α). Cronbach's alphas represent the internal consistency of the indices included in the model. Click here for file
