# Supplementary material for: Does transition from an unstable labour market position to permanent employment protect mental health? Results from a 14-year follow-up of school-leavers
Source: BMC Public Health. 2008 May 13;8:159. doi: 10.1186/1471-2458-8-159 (PMC2409329; doi:10.1186/1471-2458-8-159)
Supplement: Additional file 1 — Correlations between the variables labour market position, health-related selection, possible confounders and mediators among men (below the empty cells and italics) and women (above the empty cells). The data provided represent correlations between the independent variables. [file 1471-2458-8-159-S1.doc]

| **Additional file 1. Correlations between the variables labour market position, health–related selection, possible confounders and mediators among men (below the empty cells and italics) and women (above the empty cells).** | | | | | | | | | | | | | | | | |
| --- | --- | --- | --- | --- | --- | --- | --- | --- | --- | --- | --- | --- | --- | --- | --- | --- |
|  | |  |  |  |  |  |  |  |  |  |  |  |  |  |  |  |
| Construct | | 1 | 2 | 3 | 4 | 5 | 6 | 7 | 8 | 9 | 10 | 11 | 12 | 13 | 14 | 15 |
| 1. Labour market position (25–30) | |  | *0.060* | *0.223*** | *-0.071* | *0.074* | *0.141*** | *0.000* | *0.059* | *-0.202*** | *-0.200*** | *0.090* | *-0.120** | *0.055* | *0.344*** | *0.121** |
| 2. Presence of psychological symptoms (16) | | *0.120** |  | *0.040* | *0.051* | *0.068* | *0.105** | *0.012* | *0.088** | *-0.047* | *0.176*** | *-0.043* | *-0.030* | *0.040* | *0.093** | *0.140*** |
| 3. Unemployed at young age (16–21) | | *0.204*** | *0.049* |  | *0.086* | *0.224*** | *-0.036* | *0.267*** | *0.053* | *-0.063* | *-0.087* | *0.175*** | *-0.193*** | *0.040* | *0.093** | *0.089** |
| 4. Have children (21) | | *-0.045* | *-0.049* | *0.112*** |  | *0.124*** | *0.003* | *0.213*** | *0.102** | *0.013* | *0.056* | *0.119** | *-0.051* | *0.181*** | *0.093** | *-0.004* |
| 5. Financial problems (21) | | *0.145’’* | *0.029* | *0.160’’* | *0.144*** |  | *0.022* | *0.169*** | *0.133*** | *0.013* | *0.056* | *0.119** | *-0.051* | *0.011* | *0.029* | *0.173*** |
| 6. Unemployed relatives (21) | | *0.063* | *0.003* | *0.073* | *-0.041* | *0.001* |  | *0.047* | *0.025* | *-0.014* | *-0.030* | *0.009* | *-0.014* | *-0.002* | *0.074* | *0.109** |
| 7. Blue-collar worker (30) | | *0.115** | *0.024* | *0.182*** | *0.079* | *0.159*** | *0.141*** |  | *0.233*** | *-0.003* | *0.035* | *0.288*** | *-0.140*** | *-0.007* | *0.145* | *0.093** |
| 8. Do not do what I want to (30) | | *0.141*** | *0.147*** | *0.097** | *-0.045* | *0.085** | *0.097** | *0.229*** |  | *-0.140*** | *0.077* | *0.329*** | *-0.155*** | *0.177* | *0.167*** | *0.191*** |
| 9. High WIS (30) | | *-0.175*** | *-0.035* | *-0.140*** | *-0.110** | *-0.031* | *0.229*** | *-0.084** | *-0.0.90** |  | *0.116*** | *0.129*** | *0.097** | *-0.105** | *-0.025* | *0.044* |
| 10. High demands (30) | | *-0.177*** | *0.053* | *-0.112** | *-0.008* | *-0.053* | *-0.036* | *-0.162*** | *-0.015* | *0.113*** |  | *0.038* | *0.075* | *-0.115** | *-0.029* | *0.083* |
| 11. Low control (30) | | *0.147*** | *0.053* | *0.168*** | *-0.022* | *0.125*** | *0.109** | *0.424*** | *0.318*** | *-0.072* | *-0.136*** |  | *-0.243* | *0.185*** | *0.158*** | *0.256*** |
| 12. Poor social network (30) | | *-0.130*** | *-0.079* | *-0.064* | *0.017* | *-0.090** | *-0.015* | *-0.078* | *-0.132*** | *0.034* | *0.127*** | *-0.117*** |  | *-0.380*** | *-0.170*** | *-0.154* |
| 13. Poor social support (30) | | *0.185*** | *0.049* | *0.144*** | *-0.020* | *0.103** | *0.087* | *0.045* | *0.192*** | *-0.047* | *-0.130*** | *0.252*** | *-0.358*** |  | *0.047* | *0.246*** |
| 14. Risk of unemployment (30) | | *0.362*** | *0.149*** | *0.219* | *0.005* | *0.080* | *-0.018* | *0.152*** | *0.167*** | *-0.033* | *-0.084* | *0.160*** | *0.156*** | *0.175*** |  | *0.254*** |
| 15. People look down on me (30) | | *0.156*** | *0.110** | *0.182*** | *-0.004* | *0.099** | *0.039* | *0.130*** | *0.180*** | *-0.057* | *0.020* | *0.245* | *-0.194*** | *0.145* | *0.248*** |  |
| ** P-value <0.01  * P-value <0.05 |  | | | | | | | | | | | | | | | |
