# Supplementary material for: Does transition from an unstable labour market position to permanent employment protect mental health? Results from a 14-year follow-up of school-leavers
Source: BMC Public Health. 2008 May 13;8:159. doi: 10.1186/1471-2458-8-159 (PMC2409329; doi:10.1186/1471-2458-8-159)
Supplement: Additional file 2 — The internal consistency for all indices included in the model measured by Cronbach's alpha (α). Cronbach's alphas represent the internal consistency of the indices included in the model. [file 1471-2458-8-159-S2.doc]

Additional file 2. The internal consistency for all indices included in the model measured by Cronbach’s alpha (α).

| Index | α |
| --- | --- |
| Presence of psychological symptoms (16) | 0.83 |
| Presence of psychological symptoms (30) | 0.78 |
| High WIS (30) | 0.84 |
| High demands (30) | 0.76 |
| Low control (30) | 0.53 |
| Poor social network (30) | 0.78 |
| Poor social support (30) | 0.66 |
